# Determinants of the quality of life of care partners in the context of surgical cardiovascular interventions: A qualitative study

**DOI:** 10.1371/journal.pone.0341568

**Published:** 2026-01-27

**Authors:** Parmis Mirzadeh, Eric M. Horlick, Maral Ouzounian, Mark Osten, Miranda Witheford, Rima Styra

**Affiliations:** 1 York University, Toronto, Ontario, Canada; 2 Peter Munk Cardiac Center, University Health Network, Toronto, Ontario, Canada; 3 Department of Psychiatry, University of Toronto, Toronto, Ontario, Canada; 4 Center for Mental Health, University Health Network, Toronto, Ontario, Canada; Cardiff University, UNITED KINGDOM OF GREAT BRITAIN AND NORTHERN IRELAND

## Abstract

**Introduction:**

Cardiovascular disease (CVD) remains the leading cause of death globally, and numerous patients undergoing cardiovascular surgery rely heavily on informal care partners, often spouses or close family members for support. While much of the existing literature focuses on caregiver burden, particularly in dementia-related conditions, less is known about the specific factors influencing the quality of life (QoL) of care partners in the context of surgical cardiovascular interventions.

**Methods:**

This qualitative study was conducted at a tertiary cardiac center to identify the key factors that influence the QoL of care partners of patients who have undergone or are undergoing cardiovascular surgery. Twenty care partners of cardiac or vascular patients participated in semi-structured interviews. Interviews explored emotional, physical, financial, and social dimensions of the care partner’s experience. Thematic analysis was used to identify recurrent factors influencing care partner QoL.

**Results:**

Participants were predominantly female (80%) and most often spouses of the patients (55%). Thematic analysis revealed several interrelated domains affecting care partner QoL: financial stress (e.g., lost income, travel costs), emotional impact, communication with healthcare teams, care partner physical health limitations, and availability of social support. Patient-related factors such as the type and urgency of surgery, post-operative condition, and patient attitude also significantly influenced care partner experiences. These findings highlight the multifaceted nature of care partner QoL and its direct connection to patient outcomes.

**Conclusions:**

This study identifies core factors that impact the well-being of care partners of cardiovascular patients and emphasizes the importance of their role in supporting recovery. The results provide a framework for identifying at risk care partners for poor patient outcomes, and consideration of early intervention and tailored support. Integrating care partner needs into healthcare planning may improve both patient and care partner outcomes in cardiovascular surgical care settings.

## Introduction

Cardiovascular disease (CVD) is the leading cause of death globally [1], affecting nearly half the adult population [2]. A broad range of cardiovascular surgery is available, varying significantly in terms of invasiveness, risk of complications, surgery duration, and post operative care requirements. Individuals living with CVD often rely on informal care givers, now more commonly referred to as “care partners”, to support their treatment course and self-care [3].

Care partners provide essential, informal assistance to individuals with a health condition, which may or may not involve direct physical care, and often participate in the patients’ health-related discussions [4]. In the context of CVD interventions, care partners play a critical role before, during, and after treatment, making their involvement and contribution a key factor to consider in patient care. This issue affects a substantial portion of the patient population. For instance, in 2018, Statistics Canada reported 7.8 million individuals aged 15 or older provided care to a family member or friend living with a chronic health condition [5]. Similarly, in 2020, 53 million American adults were informal caregivers [6]. Care partners play an important role in reducing the burden on the healthcare system as they may reduce the need for hospitalization, formal caregivers, and potential admission to a long-term care facility, to name a few. Further, it is estimated that the annual savings to the American healthcare system is approximately $350 billion [7].

The level and nature of care required varies depending on the patient’s condition and symptom severity. While much of the literature has focused on caregiver burden [8–10], especially in dementia or Alzheimer’s disease, fewer studies have addressed caregiving in the cardiovascular context, and there is limited knowledge regarding care partner burden associated with cardiovascular surgery. A survey of 585 informal caregivers for patients with atrial fibrillation in the United Kingdom, Italy, and Germany was conducted and found that the average amount of time providing care per week varied (33 ± 29 hours) [11]. Further, this study found that comorbidities such as diabetes and respiratory diseases significantly contributed to time and burden [11]. While caregivers dedicate many hours to informal care of patients, many also reported fulfillment in their role [11,12]. Additionally, a meta-analysis of heart failure patients demonstrated that increased caregiver stress was significantly associated with poorer patient outcomes, including greater symptom severity, reduced patient quality of life (QoL), and increased clinical events [13].

Despite a growing body of research on caregiver burden, there remains a limited understanding of the specific factors that influence the QoL of care partners of individuals undergoing or having undergone cardiovascular surgery. QoL is a multidimensional concept that reflects the overall well-being of an individual at a point in time, encompassing both negative and positive aspects of their experience [14]. The many dimensions of QoL include physical, mental, and spiritual health, relationships, wealth, work, security, autonomy, social belonging, and physical environment, to name a few [14]. This study seeks to address this gap in the literature by identifying key determinants that impact the QoL of care partners supporting patients with CVD requiring surgical intervention. This work aims to provide an initial evidence base to identify key areas of challenges for care partners and inform the development of targeted supports specific to care partners who are supporting patients with CVD requiring surgical intervention.

## Methods

### Study design and population

This study utilized semi-structured interviews with 20 care partners at a tertiary cardiac care centre. Eligible participants for this study included care partners of patients that have undergone or were in hospital scheduled to undergo cardiovascular surgery. For the purpose of this study, care partners were defined as informal participants (e.g., family members, friends) who were not merely related or acquainted but actively involved in the patients care. For example, assisting the patient with activities of daily living, accompanying the patient to medical appointments, helping in the management of the patient’s medications, or providing ongoing emotional support, to name a few. The inclusion criteria consisted of the following: 1) at least 18 years of age, 2) care partners of patients who are hospitalized and require or have undergone cardiovascular surgery, and 3) care partner is English speaking. Participants were ineligible to participate if they were: 1) care partners with cognitive impairment, or 2) paid caregivers.

### Recruitment and data collection

Participant recruitment took place between August 11, 2023 to September 20, 2024 in the Peter Munk Cardiac Centre, University Health Network (Toronto, Canada). Potential care partners were identified during visiting hours by members of the patient’s circle of care (often nurses). Once identified, a member of the patient’s circle of care asked the care partner whether they would agree to being contacted by a member of the research team at University Health Network. For care partners who agreed, a member of the research team approached them in person to explain the study and obtain written informed consent. Potential participants were given as much time as needed to ask questions about the study and to read the consent form prior to signing. All interviews were conducted immediately following informed consent. Participants who consented to participate were taken to a meeting/conference room in the same unit at the hospital for privacy, where the interview was conducted.

A summer research student and a study author (PM) obtained consent and conducted the interviews. The semi-structured interview guide, developed by the research team, (S1 File) was used to facilitate the discussion. The guide began with a standardized introduction and definitions of key terms such as “quality of life” and “care partner” to ensure shared understanding. Participants were asked to describe their relationship to the patient, including relevant context such as the patient’s surgery and their overall involvement. The interview then progressed to broader questions exploring factors influencing the care partners’ QoL, including aspects of their mental and physical health. At the conclusion of the interview, the participants were given an opportunity to share any additional insights or experiences they felt are important for the research team to understand but may not have been addressed in the preceding questions. Given the semi-structured nature of the interviews, flexibility was maintained to allow for deviations from the general guide. This approach enables the interview to accommodate the flow of the conversation, explore emerging themes, and gain a deeper contextual understanding of participant responses. Overall, the interviews ranged from approximately twenty to sixty minutes in length.

### Data analysis

Demographic variables are summarized using descriptive statistics (frequency and percentage). Qualitative data is captured through the semi-structured interviews; therefore, thematic analyses [15] were conducted to analyze the interview responses. Two of the authors (PM and RS) independently reviewed the full documents consisting of interview answers, and identified key statements related to participants’ perceptions of QoL. Each reviewer individually coded the relevant statements, focusing on those that provided insight into factors influencing care partner QoL. The reviewers then met to compare and reflect on the key statements they identified, coding them by hand to collaboratively finalize the coding framework through consensus. This process yielded key factors affecting QoL of care partners, as well as patient-dependent factors affecting the QoL of care partners. The reviewers then revisited the transcripts to extract illustrative quotes supporting the emerging themes. The data analysis process took several meetings to discuss themes and review text, ensuring methodological rigor and integrity. Due to the study design and qualitative nature of the data, we recruited 20 participants. Although thematic saturation is commonly achieved with 12–13 participants [16], a larger sample size was obtained for robustness and thematic depth.

All study data were stored in password-protected Google Docs and Google Sheets, shared between two study authors (PM and RS) and a summer research student. Data will be deleted 10 years following the beginning of recruitment, in accordance with the institutional Research Ethics Board requirements. Interviews were not audio recorded; instead, detailed notes capturing participants responses as verbatim as possible were typed in real time during the interview. Where needed, participants were asked to repeat their sentences, to ensure completeness. No personal identifiers such as participant names or initials were collected during the interviews; therefore, quotes are presented without codes, preserving confidentiality in this small sample.

### Ethics

Ethics approval for this study was obtained from the University Health Network Research Ethics Board (CAPCR Study ID: 23–5392). All participants signed written informed consent forms to participate in the study.

## Results

Care partner and patient characteristics are shown in Table 1. Overall, 20 care partners were interviewed in this study, 80% were female, and 20% were male. Just over half (55%) were the patient’s spouse or partner, while the remaining were the patient’s children (30%), parent (5%), sibling (5%), or grandchildren (5%). Within their roles, the level of care varied, as some care partners reported only needing to attend appointments with the patients, and others reported needing to help the patient with activities of daily living. During interviews, care partners described their roles, many emphasized that accompanying patients to medical appointments and seeking clarification or additional information from healthcare providers constituted a significant part of their role.

**Table 1:** Care partner and patient characteristics.

Care Partner Characteristics	Frequency n (%)	Patient Characteristics	Frequency n (%)
**Sex**		**Sex**	
Male	4 (20)	Male	16 (80)
Female	16 (80)	Female	4 (20)
**Relationship to Patient**			
Spouse/Partner	11 (55)		
Children	6 (30)		
Parent	1 (5)		
Sibling	1 (5)		
Grandchildren	1 (5)		

All care partners were over the age of 18 years, in line with the eligibility criteria.

Key factors influencing the QoL of cardiovascular patients’ care partners are listed in Table 2, along with illustrative examples. Commonly reported challenges included financial stress due to taking time off from work and incurring costs associated with travel, parking, food, and hotel room as some care partners lived a distance from the hospital. The emotional burden associated with their roles was a significant theme, as were issues related to communication with the healthcare team (i.e., clarity of information, tone of interactions with healthcare team), the care partner’s own physical health, and the availability of supports from family and friends. Care partners valued proactive explanations from the healthcare team, which reduced the need for care partners to search for information independently. They also communicated that a cheerful bedside manner positively affected them. Many care partners reported being unable to prioritize their own health needs and frequently forgoing previously enjoyed activities, as the demands of their role occupied the majority of their time (e.g., no longer playing a sport they enjoy because they now spend time taking care of the patient). Overall, there is duality to the key factors related to the quality of life of care partners. These factors may be positive or negative, depending on the individual experience, as shown in Table 2. For example, for the key factor of physical health, while a care partner indicated no longer drinking alcohol (positive effect), another stated no longer being able to play a sport they enjoy (negative consequence).

**Table 2:** Key factors related to the quality of life of care partners of patients undergoing or having undergone cardiovascular surgery.

Factors	Explanation	Examples
Financial Stress	The financial burden associated with patients’ treatment, taking time off from work, and spending to travel to the hospital negatively impacts quality of life.	• “I was only supposed to be off work for a few months, but now it’s been over a year later and I’m honestly not really sure what my employment situation is…I don’t really know when I’ll be able to get back to it, it’s not really something I’ve even had time to think about” • “Finding it difficult to do work even remotely because I am constantly stressed about the patient” • “Life is priceless, but I’d be lying if I said the financial stress isn’t always at the back of my mind”
Emotional Impact	Care partners feel persistent worry and stress in regards to their roles, often due to the overwhelming sense of responsibility.	• “I am with him 20+ hours a day, but even those 4 hours that I’m away, I’m just constantly thinking about him and if he’s being taken care of, and I’m anxious to come back to him” • “The nurses aren’t able to check up on him as often as he needs, something could happen in a split second and if no one is there to help him then something bad could happen” • “Stress and anxiety of not knowing” • “It was crazy and surreal…our world shifted within a day, suddenly nothing else mattered” • “A scary experience with things happening quickly and having to go through so many tests and operations all of a sudden”
Communication	Quality of communication with healthcare providers and accessibility of information allows care partner to avoid taking time to do own research.	• “The doctors we spoke with were really good at explaining everything that was going on despite things being so hectic, it helped us feel kept in the loop” • “When doctors and nurses come to [the patient] and are cheerful and communicative, it creates a good impression and makes us feel more at ease with the situation” • “The doctors were really confident and explained things clearly, they’ve clearly competent and done it many times and didn’t seem worried at all, which helped put us more at ease”
Physical Health	Being a care partner often affects the physical health, changing physical habits and health condition in either positive or negative ways.	• “When I take care of myself, I am better able to help my mother” • “Lost about 20 pounds in the past few months and no longer drink alcohol” • “Used to play tennis and be active but not anymore since patient has been sick” • “I tell him (patient) I’m going to the gym, but I have my phone on me, so it feels like I’m always there…”
Supports	Having a support system and several care partners also contributing to the care for the patient allows care partner to take breaks and feel supported.	• “Having many family members physically present, able to take turns providing support and assistance is great help” • “I feel so lucky that we have so many friends and family who care about us and are offering to help out, I can’t imagine going through if we were on our own” • “I feel very happy with family members show up and visit”

Patient-dependent factors related to the QoL of cardiovascular patients’ care partners are described in Table 3. Patient-dependent factors included surgery, with more invasive surgeries contributing to higher levels of stress; patient well-being, as care partners report better quality of life when patients were doing better; and patient attitude, where positive attitudes of patients positively affect the care partners emotional well-being.

**Table 3:** Patient-dependent factors related to the quality of life of care partners of patients undergoing or having undergone cardiovascular surgery.

Factors	Explanation	Examples
Surgery	The level of invasiveness of the surgery influences care partners levels of stress and worry.	• “To be honest, I know this is bad to say, but I really wasn’t stressed or worried all that much just because I know it’s a pretty commonly performed procedure that doesn’t really have many risks”
Patient Well-being	Care partner quality of life is dependent on the patient’s health state.	• “My quality of life is dependent on how my husband is physically feeling and how well he recovers” • “If you know the patient is doing good, everything is fine” • “[patients] stress creates stress for others” • “The surgery and patients’ health is always in the back of my mind even if I’m not in the hospital and trying to work”
Patient Attitude	Patient attitudes and motivation greatly impact care partner attitudes. If patient has a positive attitude, this positively affects care partners perception of their own well-being.	• “When he is feeling down, I try to tell him positive things, but he will just get more frustrated and say I don’t understand” • “He’s saying that he thinks hes going to die, I try to tell him to think positively but it’s hard to hear someone you love saying things like that”

**Fig 1:**
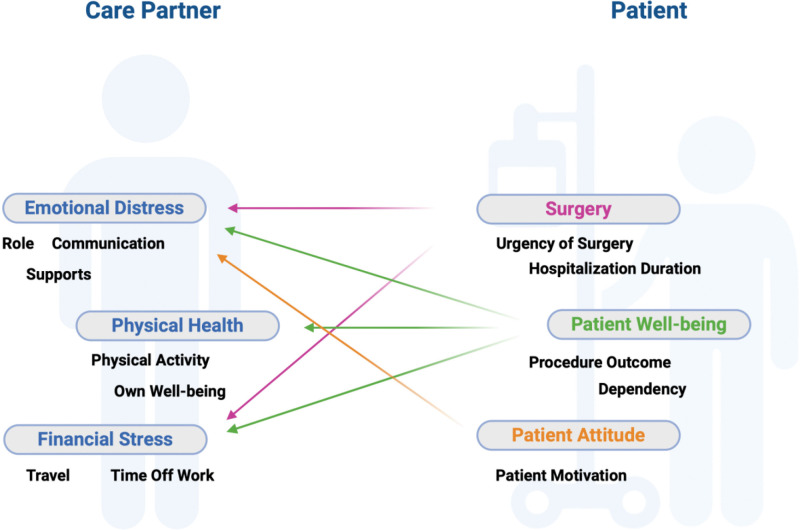
The interplay of care partner and patient dependent factors related to the quality-of-care partners of patients undergoing or having undergone cardiovascular surgery. Created in BioRender. Mirzadeh, P. (2026) https://BioRender.com/5yc52kc.

While key factors affecting care partner QoL are displayed separately, Fig 1 illustrates the dynamic interplay among them. Emotional distress was shaped by the complexity of their caregiving responsibilities and roles, the effectiveness and quality of communication with both the patient and the healthcare team, and the perceived availability and adequacy of support from family and social networks. Physical health was affected by reduced opportunities for physical activity, or pursuit of their own medical care, with several care partners reporting a decline in their own well-being. Financial stress was heightened by travelling for the surgery and often taking time off work to be there for the patient. Patient related factors shown in this figure include the surgery which is affected by both the urgency of the surgery and the duration of hospitalization, which contributed to emotional and financial strain. More complex or prolonged surgery, often required extended caregiver involvement and time off work, further contributing to overall strain. The patient’s well-being, including the clinical outcome of the surgery and level of dependency by the patient further influenced the care partner’s emotional and physical health. Finally, patient attitude encompassed the level of motivation displayed, which played a significant role in influencing the emotional distress of their care partner.

## Discussion

This study contributes to the literature by exploring the determinants influencing the overall well-being of care partners supporting hospitalized patients who have undergone or are undergoing cardiovascular surgery. In this study, key factors of care partner overall well-being impacting quality of life were identified, including financial stress, emotional impact, communication, physical health, and the availability of supports. In addition, patient-related factors, including surgery, the patient’s well-being, and patient attitudes were identified to significantly shape the care partner’s experience. In some cases, few of the themes displayed duality, where both positive and negative effects were noted.

The predominance of female care partners in this study is consistent with longstanding evidence that women are more likely to assume caregiving responsibilities across the lifespan. Women are disproportionately represented among those providing care for both children and dependent adults and are more likely to report associated psychological burdens, including fatigue, anxiety, and emotional distress [17]. This gendered caregiving pattern is particularly salient in the context of CVD, which has a higher prevalence and surgical intervention rate among men [18]. As such, it is not surprising that the majority of care partners in this study were women providing care to spouses or partners recovering from CVD-related events.

Care partner burden has been associated with increased rates of rehospitalization and poorer health outcomes among older adults [19,20]. In our cohort, care partners emphasized the importance of receiving support themselves while simultaneously providing care to the patient. Given the majority of care partners in this population are female, they may be more at risk to experiencing inadequate family support compared to their male counterparts. This lack of support is a recognized predictor of adverse health outcomes for the care partner [21]. As such, it is essential that healthcare systems and providers identify strategies to reduce the burden experienced by care partners during various healthcare interactions. The development and implementation of support measures must account for gender-specific differences, recognizing that female care partners may face distinct challenges and experiences relative to their male counterparts.

Psychological distress, including depression and anxiety, in care partners has been found to exceed levels observed in both the general population and the patients they support [22,23]. Pre-surgical anxiety in care partners of patients undergoing coronary artery bypass grafting (CABG) has been associated with elevated post-surgical cortisol levels [24], a marker of elevated stress which may impact the effective provision of care by the care partner. Increased levels of psychological distress in the care partner also produce undesirable psychological and behavioral consequences for the patient such as symptoms of anxiety and depression, and poor self-care which, in turn, contribute to increased hospitalization [25]. For patients with heart failure, care partner preparedness has been shown to significantly impact levels of care partner depression, which in turn affects both their physical and mental quality of life [26]. This finding underscores the importance of intervening early in the course of the patient and care partner’s journey undergoing cardiovascular surgery.

Care partners have a direct impact on patient outcomes, making their needs an important consideration in healthcare planning and policy. Support interventions should be tailored to the level of distress experienced. These may range from simple validation and acknowledgment of their burden to structured support groups where care partners can share experiences and access resources. For those experiencing significant emotional distress, it may be necessary to provide more specialized psychological support (e.g., structured conversations with healthcare professionals such as psychologists or psychotherapists), or financial support (e.g., consultation with social services to assist with financial organizations, and access to resources).

While this study has many strengths, including a unique sample of care partners and rich qualitative data identifying key factors influencing care partner well-being, there are some limitations which warrant mention. Experiences of care partners in this study are not generalizable to all care partners, as participants were specifically caring for patients who had undergone or were undergoing cardiovascular surgery. Further, limited demographic information was collected from care partners, including their age, socioeconomic status, social support networks, and health literacy which may have an influence on their experiences, and in turn their responses. For example, those of a lower socioeconomic status may experience greater financial burden and therefore a greater level of stress, or those with a social support network may experience less overall burden in caring for the patient. Furthermore, detailed data about the patient’s specific surgery was not collected. This may serve as a limitation as there is great variability in the invasiveness of different cardiovascular surgeries. Despite these limitations, results from this study serve as a foundational step in a better understanding of the impact of cardiovascular surgery on the care partner and provides a basis for consideration of methods for early identification of the care partner as well as interventions.

Findings from this study underscore the vital role of care partners in supporting the recovery process. Enhancing awareness of care partner needs in this population and incorporating these needs into healthcare planning may contribute to improved outcomes for both patients and care partners within cardiovascular care settings. Thus, future research should explore strategies for incorporating care partner supports into clinical practice, directly addressing care partner needs. These may include psychosocial support, targeted education, and/or financial resources which may directly target needs identified in this study. Future works should examine how addressing these needs and incorporating such supports may have long-term impact on both care partner and patient health outcomes, which can further reduce burden on the healthcare system.

## Conclusion

This study contributes novel insights to the existing literature by identifying factors that influence the QoL of care partners supporting hospitalized patients who have undergone or are undergoing cardiovascular surgery. Financial stress, emotional impact from their sense of responsibility, communication with the patient and healthcare team, care partner physical health, and level of support were the key factors affecting the QoL of care partners in this study. Furthermore, the surgery, well-being of the patient, and the patients’ attitude were patient-dependent factors which influenced the QoL of care partners. Further research is needed to further examine these factors in diverse settings with more demographic information, and to explore strategies for incorporating care partner needs in practice.

## Supporting information

S1 File Semi-structured interview guide. (DOCX)
