# Supplementary material for: Determinants of the quality of life of care partners in the context of surgical cardiovascular interventions: A qualitative study
Source: PLoS One. 2026 Jan 27;21(1):e0341568. doi: 10.1371/journal.pone.0341568 (PMC12843512; doi:10.1371/journal.pone.0341568)
Supplement: S1 File — (DOCX) [file pone.0341568.s001.docx]

**Semi-structured Interview Guide**

Preamble: Just a reminder that your **participation in this interview will be de-identified and you can stop at any time.** We will record the interview to help ensure we gather all information and then will delete the recording once transcribed. Your decision to participate is voluntary and does not affect the care the patient is receiving.

For the purpose of the interview, we will use the term ‘**care partner’** and ‘**quality of life’**. Care partner refers to individual who provides informal assistance to an individual with a health condition to help them sustain their well-being. Quality of life involved you being able to do the things that you want to do, for example complete day-to-day activities.

1. How long have you been a care partner for the patient? i.e., first time, ongoing medical issues.
2. What is important to your quality of life at present?
3. How is your ability to care for the patient impacting your day-to-day e.g., caring for dependents, caring for your own health, etc.
4. How is caring for the patient impacting your work, employment or social activates? i.e., taking time away from work, time away from hobbies, etc.
5. Do you find yourself emotionally distressed?
6. Have you shared your distress with the patient?
7. Do you find that waiting for medical treatment for the patient is impacting your quality of life?
8. Do you find that you need to engage in self-education? i.e., researching on your own, looking for your own resources?
9. Do you have a good support group (i.e., family/friends) or resources available?
10. Has caring for the patient impacted your relationship with them or others?
11. Has caring for the patient presented any economic burden i.e., financial stress?
12. In your opinion, how is the patient actually doing and how is this affecting your quality of life?
13. Is there anything else you would like to share with us about your experience as a care partner?
